# Quantitative phenotyping and evaluation for lettuce leaves of multiple semantic components

**DOI:** 10.1186/s13007-022-00890-2

**Published:** 2022-04-25

**Authors:** Jianjun Du, Bo Li, Xianju Lu, Xiaozeng Yang, Xinyu Guo, Chunjiang Zhao

**Affiliations:** 1 Beijing Key Lab of Digital Plant, Research Center of Information Technology, Beijing Academy of Agriculture and Forestry Sciences, Beijing, China; 2 Beijing Key Laboratory of Agricultural Genetic Resources and Biotechnology, Beijing Agro-Biotechnology Research Center, Beijing, China

**Keywords:** Lettuce leaf, Phenotyping, Vein architecture, Semantic segmentation, Data normalization

## Abstract

**Background:**

Classification and phenotype identification of lettuce leaves urgently require fine quantification of their multi-semantic traits. Different components of lettuce leaves undertake specific physiological functions and can be quantitatively described and interpreted using their observable properties. In particular, petiole and veins determine mechanical support and material transport performance of leaves, while other components may be closely related to photosynthesis. Currently, lettuce leaf phenotyping does not accurately differentiate leaf components, and there is no comparative evaluation for positive-back of the same lettuce leaf. In addition, a few traits of leaf components can be measured manually, but it is time-consuming, laborious, and inaccurate. Although several studies have been on image-based phenotyping of leaves, there is still a lack of robust methods to extract and validate multi-semantic traits of large-scale lettuce leaves automatically.

**Results:**

In this study, we developed an automated phenotyping pipeline to recognize the components of detached lettuce leaves and calculate multi-semantic traits for phenotype identification. Six semantic segmentation models were constructed to extract leaf components from visible images of lettuce leaves. And then, the leaf normalization technique was used to rotate and scale different leaf sizes to the “size-free” space for consistent leaf phenotyping. A novel lamina-based approach was also utilized to determine the petiole, first-order vein, and second-order veins. The proposed pipeline contributed 30 geometry-, 20 venation-, and 216 color-based traits to characterize each lettuce leaf. Eleven manually measured traits were evaluated and demonstrated high correlations with computation results. Further, positive-back images of leaves were used to verify the accuracy of the proposed method and evaluate the trait differences.

**Conclusions:**

The proposed method lays an effective strategy for quantitative analysis of detached lettuce leaves' fine structure and components. Geometry, color, and vein traits of lettuce leaf and its components can be comprehensively utilized for phenotype identification and breeding of lettuce. This study provides valuable perspectives for developing automated high-throughput phenotyping application of lettuce leaves and the improvement of agronomic traits such as effective photosynthetic area and vein configuration.

**Supplementary Information:**

The online version contains supplementary material available at 10.1186/s13007-022-00890-2.

## Background

The morphogenesis and growth of lettuce leaves have complex regulation mechanisms. In addition to genetics, habitat can also influence the variation in their leaf structure, color, and venation. Explainable and highly distinguishable features for large-scale lettuce varieties are very important for the applications of phenotype identification and phenotype–genotype association analysis. It is impossible to find two identical leaves worldwide [1], but the leaves of different lettuce varieties are likely to show higher similarity. Obviously, the statistical traits of the whole leaf are insufficient to describe detailed differences among varieties. Visually, lettuce leaf can be divided into different functional regions, such as petiole and veins for mechanical support and material transport, and other regions for photosynthesis. Therefore, detailed structure phenotyping of lettuce leaves can provide more abundant traits for leaf classification and identification and a more accurate computation basis for leaf functional analysis.

The basic structure of leaves is established in the early stage of leaf development. Leaf morphogenesis includes the formation of the petiole, mid-rid, lamina, and marginal structures [2]. These internal components of the leaf can be characterized and used for leaf classification, identification, grading, and functional analysis. However, it is challenging to obtain these traits manually. Image-based phenotyping can provide an automated, non-destructive, and cost-effective method for quantifying lettuce plant/leaf traits. At the plant population level, unmanned aerial vehicle (UAV) has been used to evaluate the genetic diversity in red leaf lettuce germplasm [3], identify agronomic characteristics and carotenoid-rich genotypes [4], and measure yield-related phenotypes from ultra-large aerial imagery [5]. At the plant level, high-resolution images of individual lettuces can be used to extract and evaluate more complex traits, such as aboveground biomass [6], leaf canopy area, color, texture [7–9]. But leaf occlusion in lettuce canopy or side image makes it difficult to extract complete leaf structure [10]. Besides visible imaging, hyperspectral and chlorophyll fluorescence imaging have been used to assess the deterioration of freshly-cut lettuce leaves [11]. Moreover, other imaging modalities (transmission scanning [1], X-ray imaging [12], and microscope [13], etc.) can be used to capture leaf images for segmentation and analysis of leaf veins. Relatively, visible images of detached leaves reflect much information of leaves from color to vein configuration [14].

From an image processing perspective, data-driven image techniques such as convolutional neural networks (CNNs) and their derived models can be used for the segmentation and classification of vegetable plants/leaves [15, 16]. CNN-based models can be used to detect single or multiple objects in leaves [17, 18] and conduct image-level classification [19, 20] based on accurately annotated images [21, 22]. Once leaf components, such as mid-rid, vein venation, and petiole, are extracted and quantified, more detailed information can be used to describe the characteristics of lettuce leaves. For each leaf component, image-based features can be described using its morphology [23, 24], color [25, 26], and structure [1, 14] properties. For the entire leaf, the venation and color indicate valuable biological and physiological properties. For example, various types of venation represent different tolerance to hydraulic damage [27], and several special features, such as vein density, vein branching, and the area ratios with blade area, are probably related to its photosynthetic capacity and fresh weight [28]. These vein features can also be used to investigate the effect of environmental factors on vein patterns or compare venation patterns of various species for evolutionary studies [29]. Moreover, the colors of lettuce leaves are significantly different due to the accumulation of anthocyanins via genes controlling leaf color [30]. Traditional studies focused on quantifying whole leaf color but did not distinguish color between positive and back leaves. Leaf color is substantially different between positive-back images of leaves, reflecting different physiological functions of leaves. To the best of our knowledge, leaf components and their trait differences between the positive-back leaves have not been quantitatively assessed.

This study aimed to develop an automated phenotyping pipeline to quantify detached lettuce leaves and evaluate the multi-semantic traits for large-scale lettuce cultivars. The study highlights the efficacy of an automated phenotyping pipeline in recognizing multiple semantic components of lettuce leaves and evaluating traits differences not only for leaf components but between positive and back leaves.

## Materials and methods

### Image acquisition

**Fig. 1:**
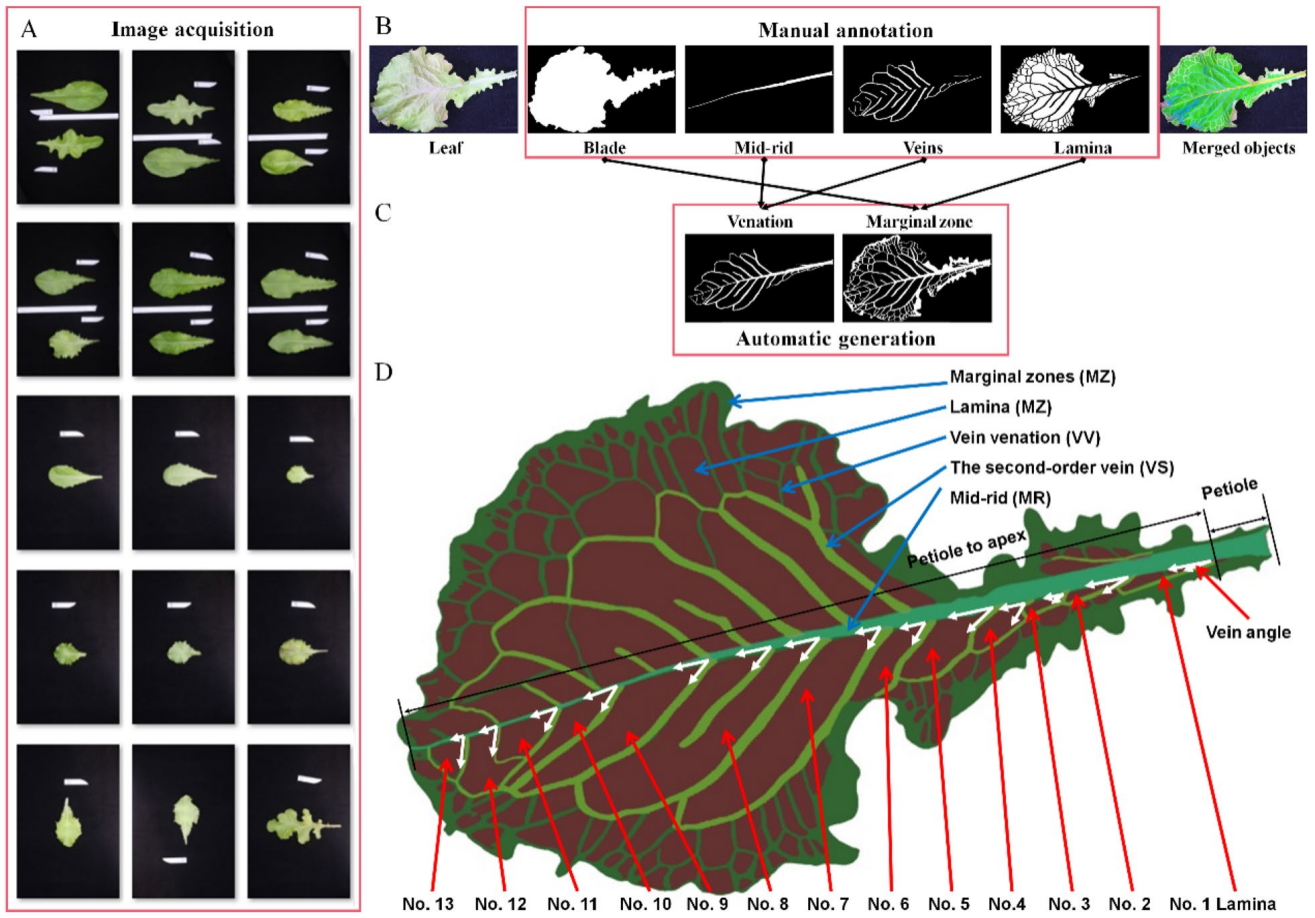
Image annotation specification for semantic components of lettuce leaves. **A** Images of lettuce leaves captured in an actual experimental environment. **B** The delineated leaf as four semantic components (*BD* blade, *MR* mid-rid, *VS* veins, and *LM* lamina). **C** Two new semantic components (*VV* venation and *MZ* marginal zone) automatically generated based on the corresponding semantic components. **D** Data annotation specification of six semantic components

More than 400 lettuce varieties were grown in a terraced greenhouse at the Beijing Academy of Agriculture and Forestry Sciences (BAAFS) from January 1 to February 25, 2020. Lettuce varieties included Butterhead, Celtuce love, Italian, Red Coral, Red Lettuce, Red Oakleaf, and Salad Grand Rapid (SGR) [31]. A studio section with a background cloth, a tripod, and a digital camera (77D, Canon Inc., Tokyo, Japan), was set up near the plants in the greenhouse for the photography. A 22 mm prime lens was used to obtain the images at a distance of about 1.2 m. A white label paper was placed near the leaf to mark the object’s size. During data collection, one representative leaf was cut from each lettuce cultivar and immediately used to acquire its positive-back images. In the procedure of image acquisition, it was challenging to ensure the consistency of leaf position and orientation (Fig. 1A). Therefore, these leaves need to be automatically detected and normalized for the subsequent phenotypic analysis, such as cropping regions of interest rotating leaf according to the positions of tip and petiole.

### Semantic segmentation

As shown in Fig. 1B, the visible components of the lettuce leaf included the petiole, mid-rid, veins, and lamina. Thus, the structure of lettuce leaves were annotated as four types of semantic components: blade (BD), mid-rid (MR), veins (VS), and lamina (LM). Two new semantic components were automatically generated based on these annotated semantic components (Fig. 1C). Venation (VV) was defined as a union set between MR and VS, and a marginal zone (MZ) was defined as a different set among the above semantic components (BD–VV–LM). Among them, MR divided the leaf into two parts (the left and right), and could be divided into petiole and first-order veins (from petiole to apex). VS (the second-order and smaller veins) were the vascular structures that transport water and photosynthetic materials. MR and VS divided the leaf into lots of flat-gridded laminas. The second-order veins and the first-order laminas had a corresponding relationship along with the MR from petiole to apex (vein and lamina orders). The data annotation specification is shown in Fig. 1D.

**Fig. 2:**
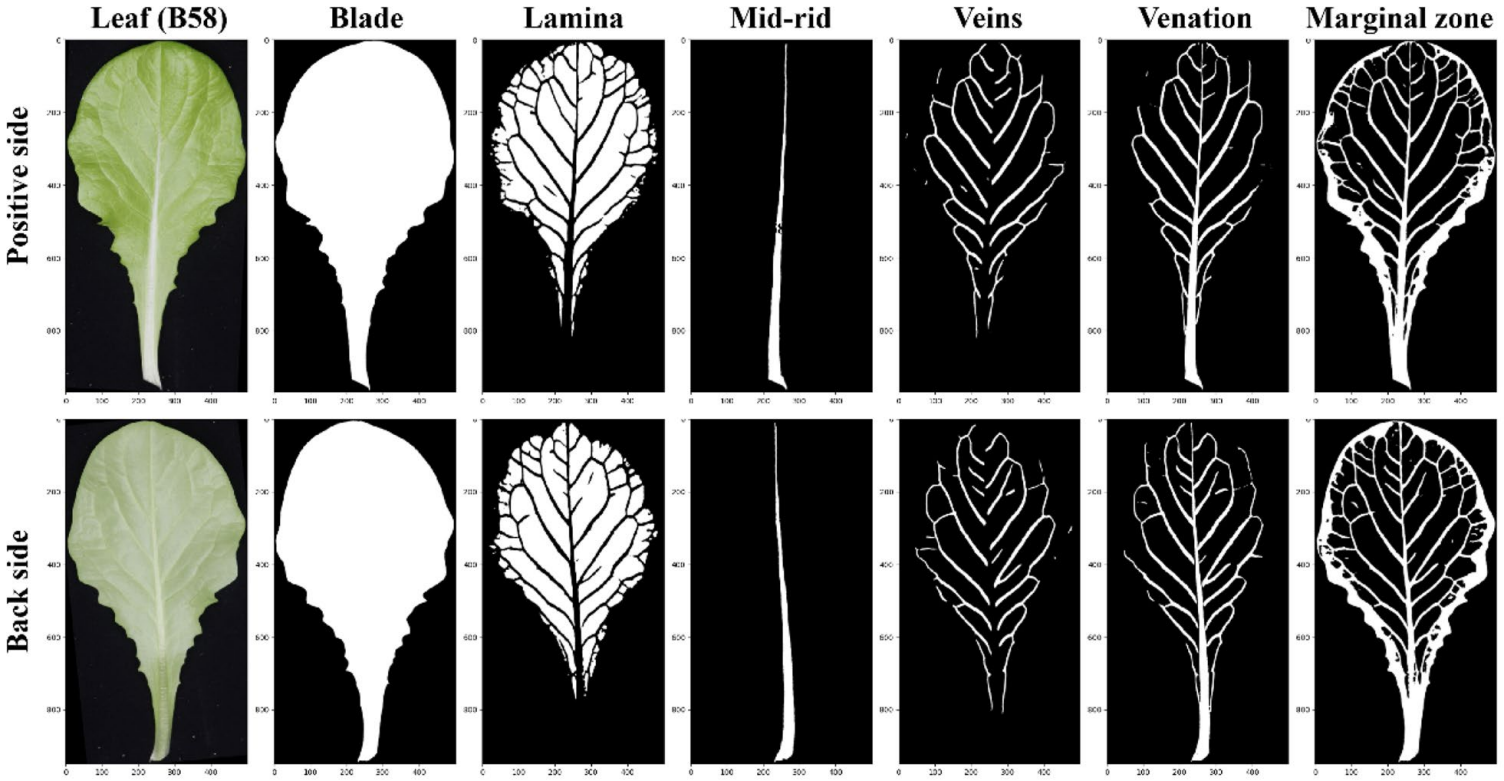
Six semantic components of positive and back lettuce leaf based on the classical U-NET models

The classical U-NET [32] was used separately to train semantic segmentation models for six components due to its good performance and adaptation for small data sets. A set of image augmentation techniques [33] was applied to increase the number of leaf instances and decrease model over-fitting. Each annotation data set was randomly divided into training, validation, and testing sets (7:2:1). The training and validation sets were used to train the model, and the testing set was used to evaluate the performance of the model. Based on these semantic segmentation models, six semantic components could be extracted from the positive and back images of lettuce leaves and used for the subsequent phenotypic analysis (Fig. 2).

### Normalization and semantic representation

Lettuce leaves could be represented using size-related and size-free traits. The size-related traits of lettuce leaf consist of common geometry indices, such as leaf area, perimeter, length, and width. These traits were calculated based on the resulted semantic components from the end-to-end semantic segmentation. However, leaves of different lettuce varieties had significant differences in size and leaf area at the same growth period. Thus, these leaves were rotated to the same orientation and scaled to the same size (width) to eliminate the effect of the size difference and improve the efficiency of the phenotyping process.

**Fig. 3:**
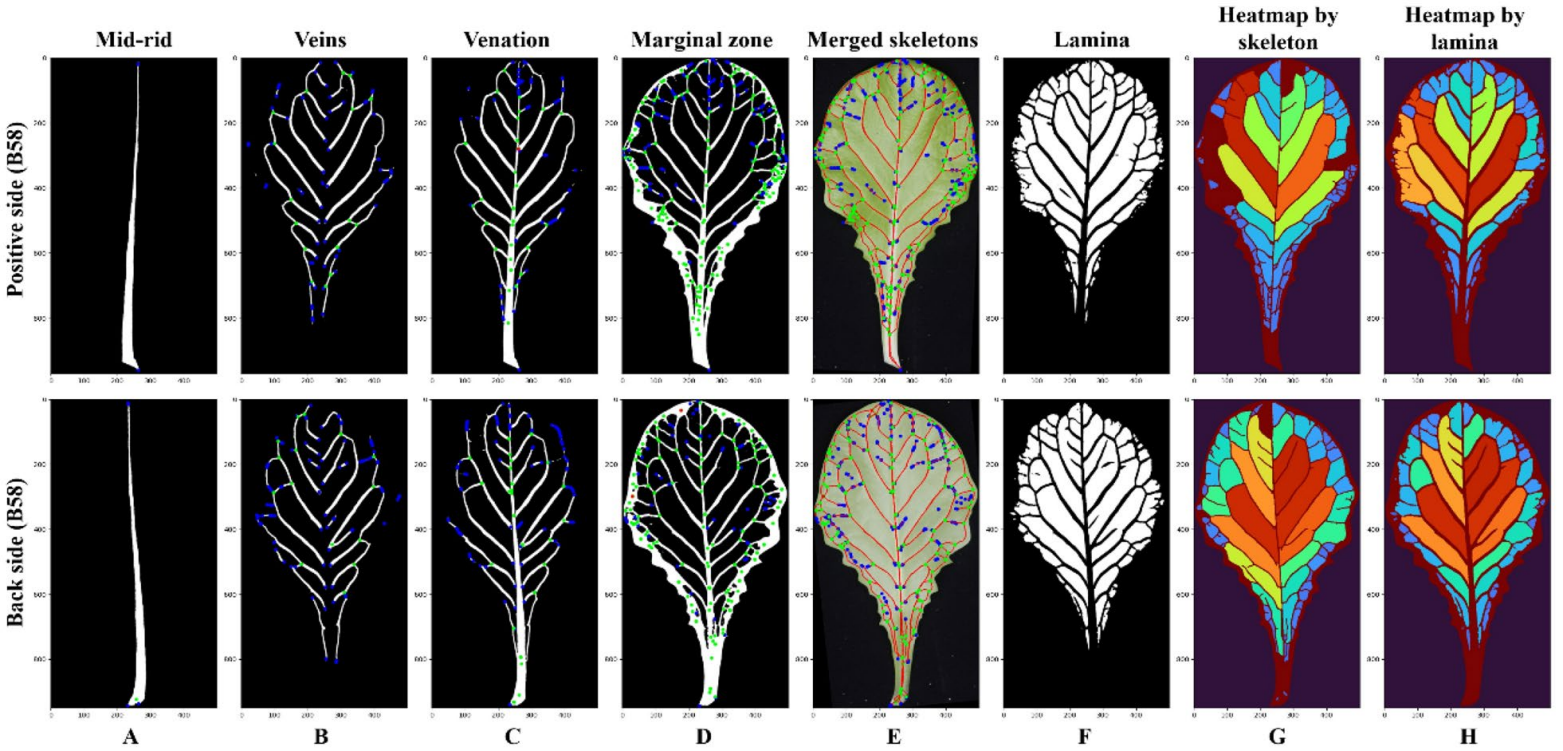
The normalized semantic components and the skeleton-based visualization of vein architecture. **A**–**D** Semantic components (mid-rid, veins, venation, and marginal zone) and their skeleton representations. **E** The merged skeletons of four semantic components. **F** Semantic component of the lamina. **G** Heatmap of individual regions divided by skeletons. **H** Heatmap of laminas

Leaf normalization was performed to obtain standard “size-free” images of lettuce leaves via rotation and scaling procedures. The leaf was rotated to an upright position according to its mid-rid (petiole and apex at the bottom and top of the image, respectively) (Fig. 3A). It could be seen that the mid-rid was curved, and the width of the petiole was always large. Thus, the petiole and apex regions of the mid-rid were identified as follows: first, the OBB (oriented bounding box) of the mid-rid was computed, then divided into two rectangles by cutting the two longer edges of OBB. Further, pixel numbers of the mid-rid were calculated for the two rectangles, and the region with a larger area was identified as the petiole region. Finally, the centroids of the petiole and apex were calculated and connected into a straight line which was taken as the reference line for leaf rotation. Size normalization was used to proportionally scale the leaf to the same width with a size-dependent scaling factor (the ratio of the original leaf width to the normalized width); thus, the normalized leaves had the same width and different lengths. In this study, the leaf width was set as 500 pixels to achieve a balance between computational efficiency and accuracy.

Four semantic components (mid-rid, veins, venation, and marginal zone) of positive and back leaves could be skeletonized (Fig. 3A–D). The endpoints and intersections of each skeleton were used to describe the vein network (marked using blue and green solid circles, respectively). These skeletons had a high overlapping degree and were merged to the original leaf image to demonstrate the consistency of veins (Fig. 3E). The skeletons divided the whole blade into lots of individual regions. An equivalent colormap was applied to visualize the leaf structure according to the area of each region (Fig. 3G). The laminas could also be colored (Fig. 3F) and were very similar to Fig. 3G. This suggested that the lamina-based method could be used to describe and assess vein architecture.

### Phenotyping of lettuce leaves

**Fig. 4:**
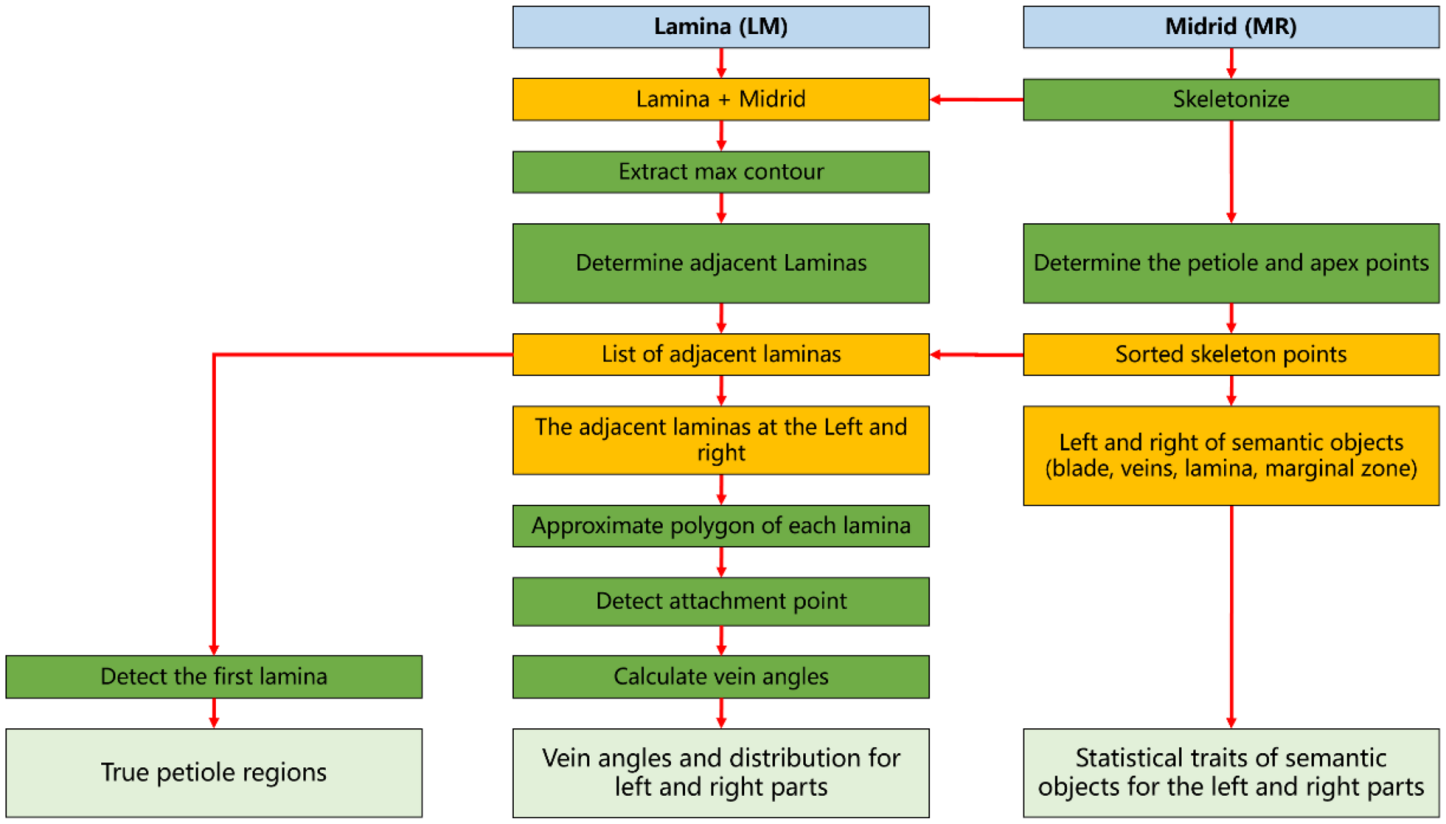
The flow chart of the phenotyping pipeline for lettuce leaves

Geometry and color traits of each semantic component could be calculated directly using semantic segmentation results. However, more valuable descriptions could be extracted from these semantic components, such as the petiole and vein architecture. The phenotyping pipeline of lettuce leaves was proposed to calculate the petiole, vein architecture, and related statistical traits, as shown in Fig. 4.

The petiole was defined as the region along the mid-rid from the leaf base to the first lamina; thus, the mid-rid could be divided into the petiole and first-order veins. The laminas and mid-rid could be combined into a new object by a dilate operation of the mid-rid region. The max contour of the new object could contain all adjacent lamina regions, and the lamina closest to the leaf base was used to determine the petiole. Moreover, the mid-rid could divide the whole leaf into the left and right parts. Thus, the skeleton line of the mid-rid was utilized to divide semantic components (blade, veins, lamina, marginal zone) into two parts (the left and right parts).

**Fig. 5:**
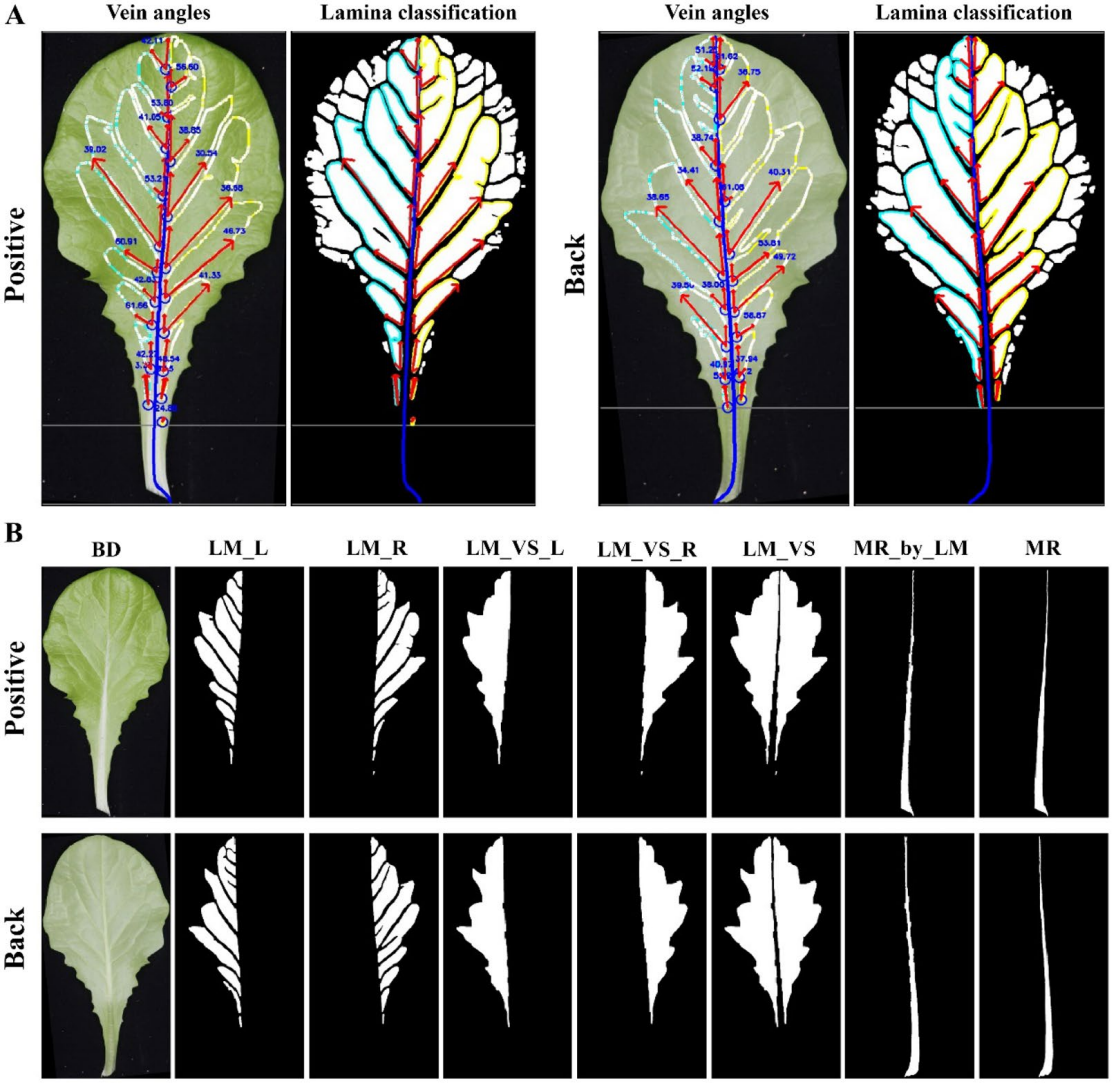
The lamina-based approach for vein architecture analysis. **A** Lamina classification and vein angle representation. **B** Lamina analysis. Blue lines indicate the skeleton line segments of the mid-rid; blue circles represent attachment points; two red arrows from each attachment point represent vein angle, and angle values are shown at the node furthest from the mid-rid; grey lines indicate the position of the petiole

The vein architecture mainly contained the angles and distribution of the second-order veins. In this study, the lamina-based approach was used to analyze vein architecture (Fig. 5A). All laminas adjacent to the mid-rid were taken as the first-order laminas, which were used to calculate the angle and direction of the corresponding veins. In Fig. 5B, the first-order laminas could be divided into LM_L and LM_R (left and right) and further merged as two parts (LM_VS_L and LM_VS_R) using morphology operations. Obviously, LM_VS regions contained both the first-order LM and second-order VS and could be used to calculate a new mid-rid (MR_by_LM) which was highly consistent with the segmentation result of the mid-rid (MR). This indicated that laminas could effectively represent the vein architecture.

Vein angles could be calculated based on the first-order laminas (LM_L and LM_R). The contour of each lamina was simplified as a polygonal chain using fewer points based on the Douglas–Peucker algorithm. The approximated polygon retained the original contour shape (controlled by the specified tolerance), and fewer nodes were used to eliminate the ambiguity in angle calculation. The simplified polygon had a subset of the points of the original contour. Due to the normalized leaves, a default tolerance, such as five, could be applied to almost all the laminas. Among the polygon nodes, the closest point to the leaf base and mid-rid was selected as the appropriate attachment point. The corresponding vein angle of each attachment point was calculated using its two adjacent line segments of the polygon.

The complex shape and structure of the lettuce leaves, such as leaf folds, twisted veins, multiple main veins, etc., presented challenges to the leaf phenotyping. For these particular leaf cases, it was difficult to extract leaf semantic components accurately by image techniques. The phenotyping pipeline could only be applied for the leaf types that conformed to data annotation specification (Fig. 1D), not for all leaf types. Thus, lettuce leaf images needed to be manually screened to ensure their validity. Moreover, there are always similar semantic components between the positive and back images of the lettuce leaf. Therefore, the positive-back images could be used not only to verify the reliability of the phenotyping methods but also to analyze trait differences between positive-back leaves.

## Results and discussion

### Evaluation of semantic segmentation

Since data annotation was highly time-consuming and labor-intensive, only 41 representative lettuce leaves were annotated based on the specification in Fig. 1D. Six semantic segmentation models (i.e., BD, VV, MR, VS, LM, and MZ) were trained respectively for 200 epochs using the same backbone and parameters (Learning rate: $5\times {10}^{-5}$, batch size: 4, backbone: inception-ResNet-v2) to achieve convergence. The Intersection over Union (IoU) and Loss of the training and validation sets are listed in Additional file 1: Table S1. IoU scores of blade, lamina, and mid-rid models achieved over 90%, but that of veins was as low as 70%. For the testing data set, the average IoU (mIoU) and F1 (mF1) of all semantic components demonstrated a similar performance with training and validation. Among components that were related to vein architecture (i.e. mid-rid, venation, and veins), the mid-rid’ model achieved relatively higher segmentation accuracy.

### Evaluation of data normalization

**Fig. 6:**
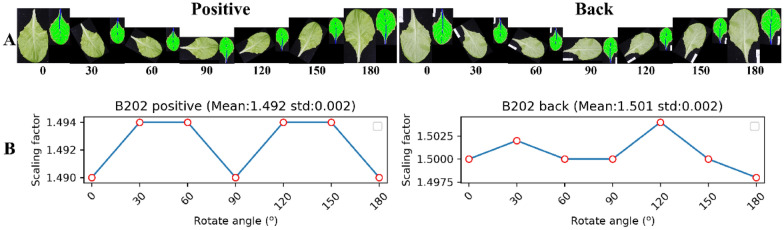
Data normalization for the positive and back leaves. **A** Rotated and normalized positive and back leaves using an angle sequence [0, 30, 60, 90, 120, 150, 180]. **B** The corresponding scaling factors. The normalized image was colored and scaled for visualization (blue and green represent the mid-rid and laminas, respectively; B202 indicates lettuce cultivar

Leaf normalization operation was an important procedure to ensure the consistency of phenotyping analysis. Each leaf image was rotated in an angle sequence [0, 30, 60, 90, 120, 150, 180] to construct test image sets which were used to evaluate the robustness and reliability of the proposed leaf normalization method. The data normalization results for the positive and back leaves are shown in Fig. 6. Mean scaling factors were very close (1.492 and 1.501) and had similar standard deviation (std) (0.002). The results indicated that the data normalization was suitable for both the positive and back images of lettuce leaves. Notably, the normalized image might need to be flipped (Fig. 6A), which was the follow-up operation of data normalization.

**Fig. 7:**
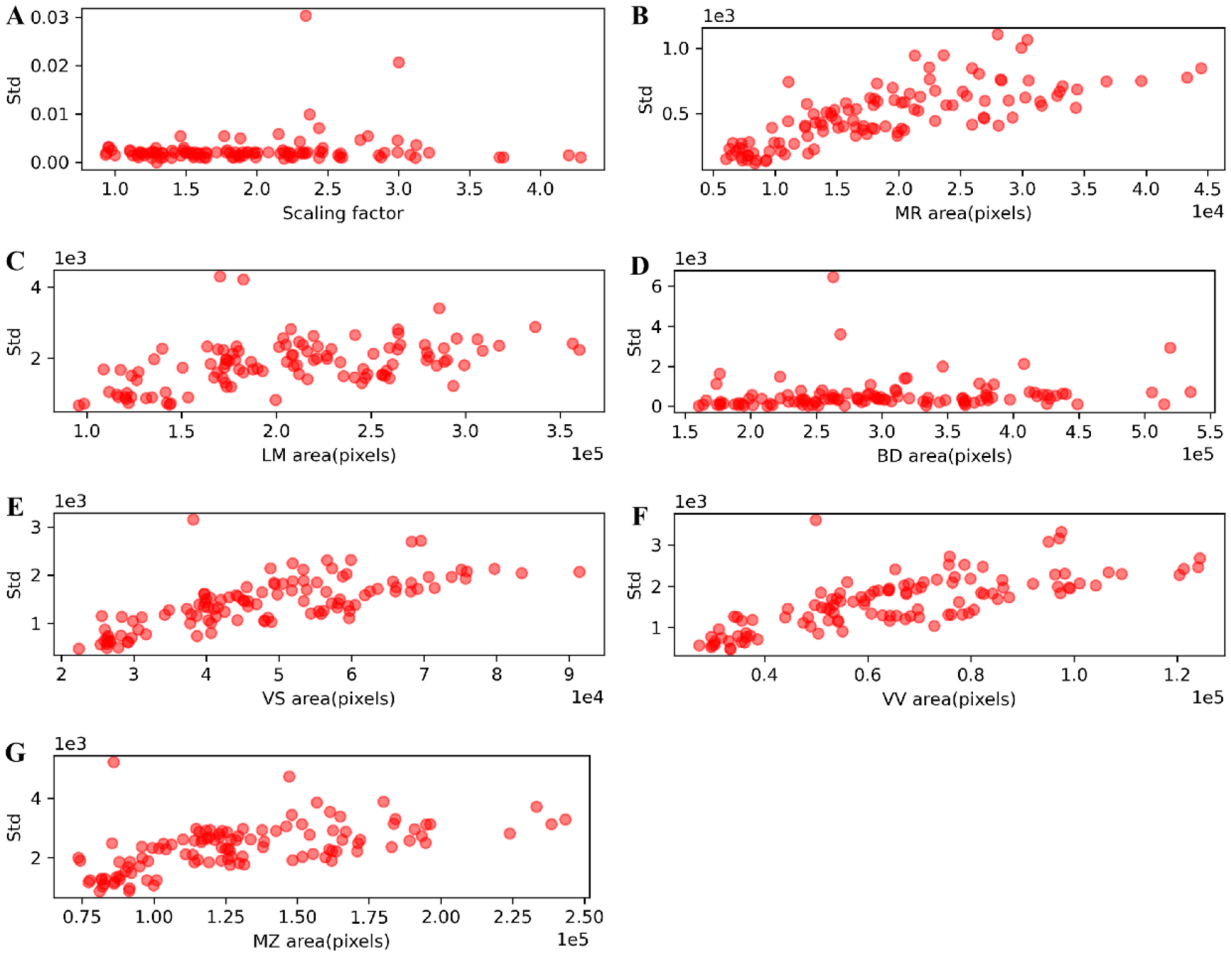
Evaluation of data normalization for 112 leaf images. **A** The deviations (Std) of scaling factors. **B**–**G** Area deviations of semantic components

The rotation invariance of data normalization could be assessed by the scaling factors. The mean and std of scaling factors were 1.949 and $3.452\times {10}^{-3}$, respectively, for 112 images of lettuce leaves, indicating that the data normalization obtained highly consistent results for leaf images. The mean and std of the rotated images were calculated for each leaf (Fig. 6B), which were then statistically analyzed (Fig. 7). The Smallest and largest scaling factors of these leaves were 0.933 and 4.283, respectively, and the standard deviation distribution of scaling factors was less than 0.03 for all the test images (Fig. 7A). The normalized images with different scaling factors had area deviations (Std). The average area deviations of six semantic components (BD, MZ, LM, VV, VS, and MR) were 781.812, 779.015, 657.497, 645.293, 523.569, 222.495 pixels, respectively (Fig. 7B–G). This suggested that the data normalization could adjust the leaves to the same orientation and had little influence on the area of each semantic component.

Leaf area rapidly increased during leaf vegetative growth, while the vein architecture (the mid-rid and second-order veins) slightly increased. Scaling factors could be used as an individual trait of lettuce leaves since they directly reflected leaf growth. Data normalization was valuable to extract size-related traits for evaluating lettuce leaves with large size differences. Moreover, data normalization greatly reduced the image size, thus improving the computation performance of the time-consuming techniques.

### Accuracy of the phenotyping pipeline

Eleven traits of 112 images from 56 lettuce leaves (each leaf contained positive and back images) were manually measured to validate the computation accuracy of the phenotyping pipeline. Manually measured traits are listed in Table 1. Three types of traits (geometry-related traits, the number of the first-order laminas, and angles of the second-order veins) were manually counted or measured using Image J software [34]. The laminas and the second-order veins were divided into two left and right parts to analyze the symmetry of vein architecture. The unit of geometry-related traits by manual measurement was pixels ($4.785\times {10}^{-2}$ mm/pixel).

**Table 1:** The traits of lettuce leaves by manual measurements

Index	Abbreviation	Description	Unit
1	BD_L	Leaf length	$\mathrm{Pixel}$
2	BD _W	Leaf width	$\mathrm{Pixel}$
3	MR_L	Length of mid-rid vein	$\mathrm{Pixel}$
4	PE_L	Length of petiole	$\mathrm{Pixel}$
5	AP_L	Length from petiole to the apex	$\mathrm{Pixel}$
6	LM_N_LT	Number of left laminas	
7	LM_N_RT	Number of right laminas	
8	LM_N	Total number of laminas	
9	LM_Ave_ANG_LT	Average vein angles of left-side leaf	$\mathrm{Degree }(^\circ )$
10	LM_Ave_ANG_RT	Average vein angles of right-side leaf	$\mathrm{Degree }(^\circ )$
11	LM_Ave_ANG	Average vein angles of the leaf	$\mathrm{Degree }(^\circ )$

**Fig. 8:**
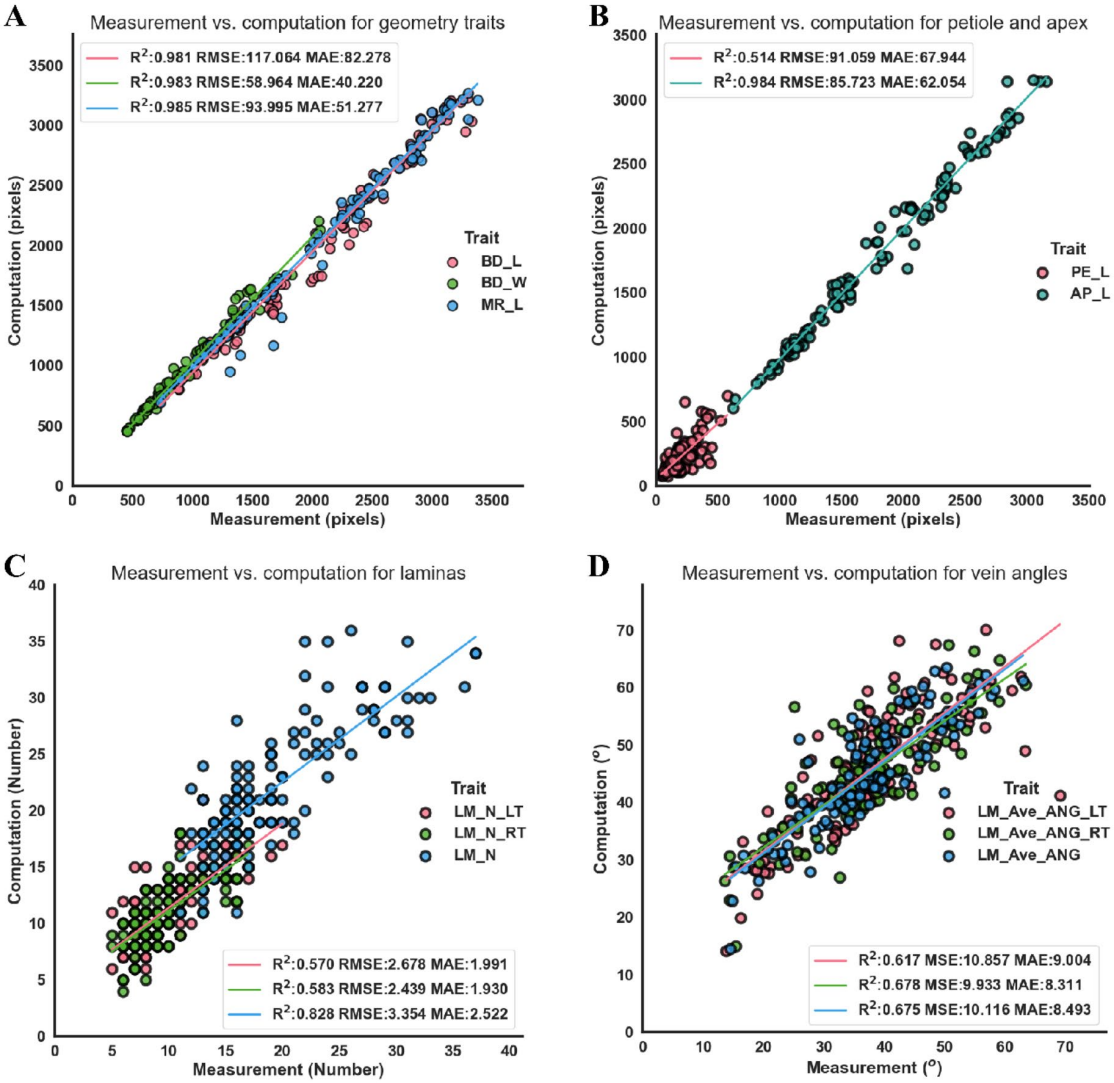
Comparison between measurement and computation traits of lettuce leaves using the scatter plots. **A** Leaf length, width, and mid-rid length. **B** Petiole length and apex length. **C** The first-order lamina number of the leaf-side, right-side, and entire leaf. **D** Average angle of the second-order veins obtained using the lamina-based approach

The comparison analysis of the 11 traits was conducted to identify the relationship between manual and computation measurements. ${R}^{2}$ (Coefficient of determination), MAE (mean absolute error) and MAPE (the mean absolute percentage error) of the manual and computation measurements were used to evaluate the consistency and accuracy of the computation method. ${R}^{2}$ was used to determine how well the computation values fit the manual measurement. Moreover, BD_L, BD_W, and MR_L were widely used in lettuce breeding and cultivation, which demonstrated strong correlations between manual and computation measurement, ${(R}^{2}$ values, 0.981, 0.983, and 0.985, respectively) (Fig. 8 A).

MR_L had a higher correlation coefficient than BD_L. PE_L was determined by the position judgment of the first lamina in manual measurement. PE_L had the least correlation between manual and computation methods (${R}^{2}$= 0. 514), possibly due to the uncertainty of petiole for both manual measurements and computation (Fig. 8B). The MAE of size-related traits ranged from 40 to 82 pixels, indicating that the prediction error varied from 1.914 mm to 3.924 mm. The number difference of the left and right laminas was about 2, and that of the total laminas was less than 3 (Fig. 8C). The counting errors mainly came from the laminas with a small area (the first and last) on the leaf. LM_N had a higher ${R}^{2}$(0.828) than LM_N_LT and LM_N_RT. This study assessed the average angle of the second-order veins (Fig. 8D). The MAE of average vein angle (8$^\circ$) was strongly correlated with ${R}^{2} ($over 0.6) between manual and computation measurements. Overall, the consistency between computed and manually-measured traits confirmed the efficacy of the digital phenotyping of lettuce leaves since several new and hard-to-measure traits could be acquired using the proposed method.

The phenotyping pipeline of lettuce leaves was further utilized to process positive and back images of all lettuce cultivars. In this study, 400 valid lettuce leaves (a leaf corresponds to a variety and contains positive and back images) were utilized to verify the accuracy of the proposed method and evaluate the trait differences between positive and back images.

### Evaluation of semantic components

**Fig. 9:**
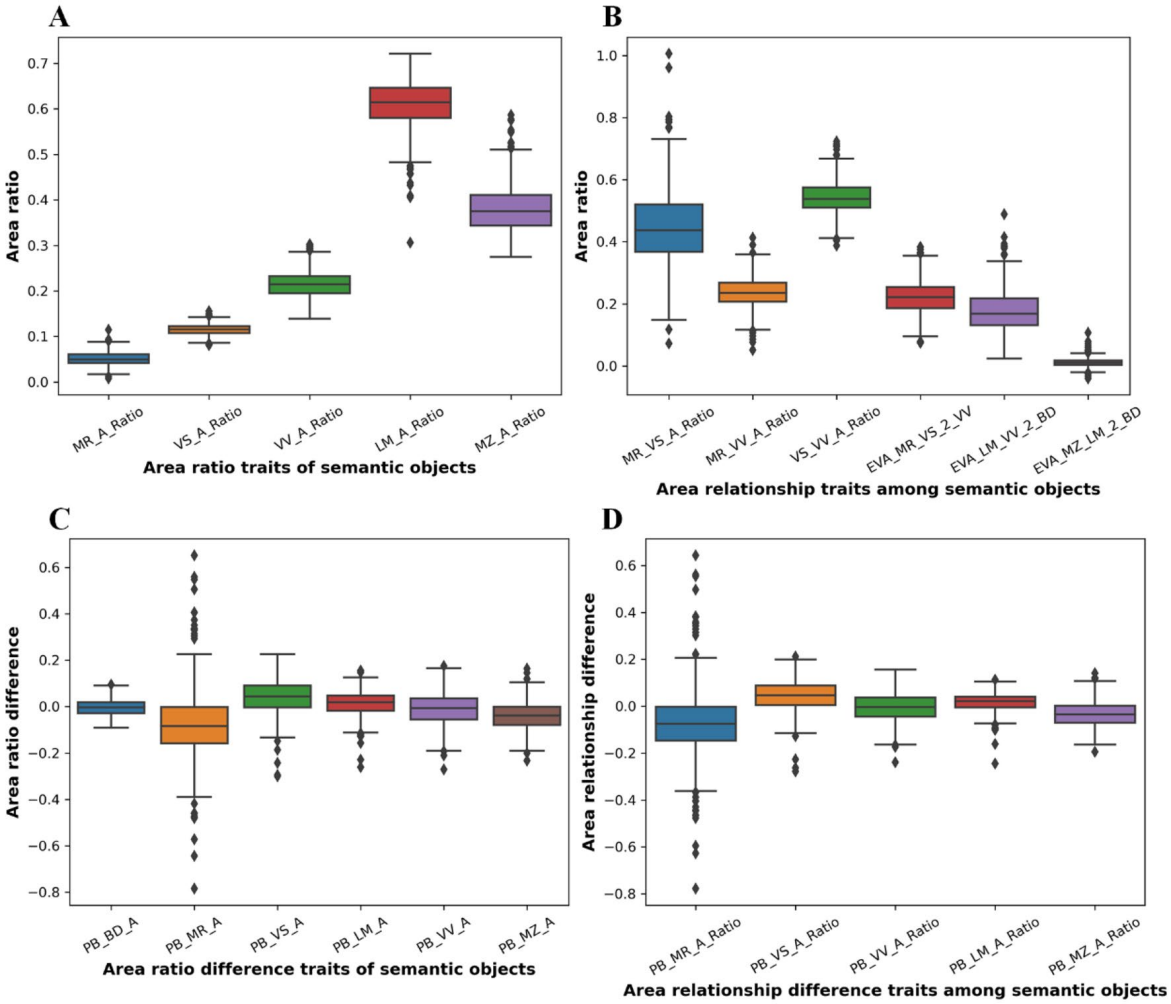
Statistical analysis for area ratio of semantic components and area relationship among semantic components. **A** Area ratio of semantic objects. B. Area relationship among semantic objects. **C** Area ratio difference between positive and back leaves. **D** Area relationship difference between positive and back leaves

The common geometry traits of lettuce leaves, such as length, width, pixel area, convex hull area, were calculated based on each semantic component after data normalization (Additional file 1: Table S2). The area proportion relationship between five semantic components (mid-rid, veins, venation, lamina, and marginal zone) and the total leaf was essential for understanding the leaf structure and components. Eight indicators were used to evaluate the area ratio of each semantic component relative to the total area of BD (Fig. 9A), i.e. MR_A_Ratio, VS_A_Ratio, VV_A_Ratio, LM_A_Ratio, MZ_A_Ratio, MR_VS_A_Ratio, MR_VV_A_Ratio, and VS_VV_A_Ratio. The average area ratios of MR, VS, VV, LM, and MZ were 5.126%, 11.493%, 21.430%, 60.939%, and 38.001%, respectively. Laminas had the largest area ratio, more than three-fifths of BD. The total area of petiole and vein venations was also more than one-fifth of BD. The total area of LM and MZ reached 98.94% of the BD. The total area of MR and VS was significantly lower than the area of VV (16.619% vs. 21.430%). Moreover, the semantic components of the lettuce leaf had overlay regions (pixels), such as (MR + VS) and VV; (LM + VV) and BD; (MZ + LM) and BD. Three indicators were used to evaluate the area relationship among semantic components, i.e. EVA_MR_VS_2_VV, EVA_LM_VV_2_BD, EVA_MZ_LM_2_BD (Fig. 9B). MR, VS, and VV had the highest overlapping ratio (EVA_MR_VS_2_VV, 22.222%), while MZ, LM, and BD had the least overlapping ratio (EVA_MZ_LM_2_BD, 1.060%).

Area ratio and area relationship of semantic components between positive and back leaves were calculated to evaluate the accuracy of semantic segmentation. PB indicates the area difference between positive and back leaves. As shown Fig. 9C, the absolute area differences between positive and back leaves for six semantic components were as follows: PB_MR_A (−7.780%) > PB_VS_A (4.050%) > PB_MZ_A (−3.983%) > PB_LM_A (1.282%) > PB_VV_A (−1.078%) > PB_BD_A (0.448%). Obviously, the semantic segmentation models had good adaptability for BD (the positive and back) but the largest error for MR (the positive and back). The area difference among five semantic components had similar results as follows: PB_MR_A_Ratio (−7.409%) > PB_VS_A_Ratio (4.449%) > PB_MZ_A_Ratio (−3.577%) > PB_LM_A_Ratio (1.735%) > PB_VV_A_Ratio (−0.651%), as shown in Fig. 9D.

The structure and the area relationship among semantic components of lettuce leaves were quantitatively evaluated. The results indicated that the segmentation models of the entire leaf, venation, lamina, and marginal zone were more robust and could obtain high consistency for positive-back leaves. The segmentation of MR and VS were relatively sensitive to positive-back leaves, therefore more vein-related semantic components were used for the subsequent evaluation of vein architecture.

### Evaluation of vein architecture

MR, VS, VV, and LM were utilized to quantitatively analyze the vein architecture of lettuce leaves based on skeleton-based (Fig. 3) and lamina-based methods (Fig. 5). The lamina-based technique was used to calculate the angles of the second-order veins due to its higher segmentation accuracy and simpler post-processing operations. The detailed traits of vein architecture are shown in Additional file 1: Table S3.

**Fig. 10:**
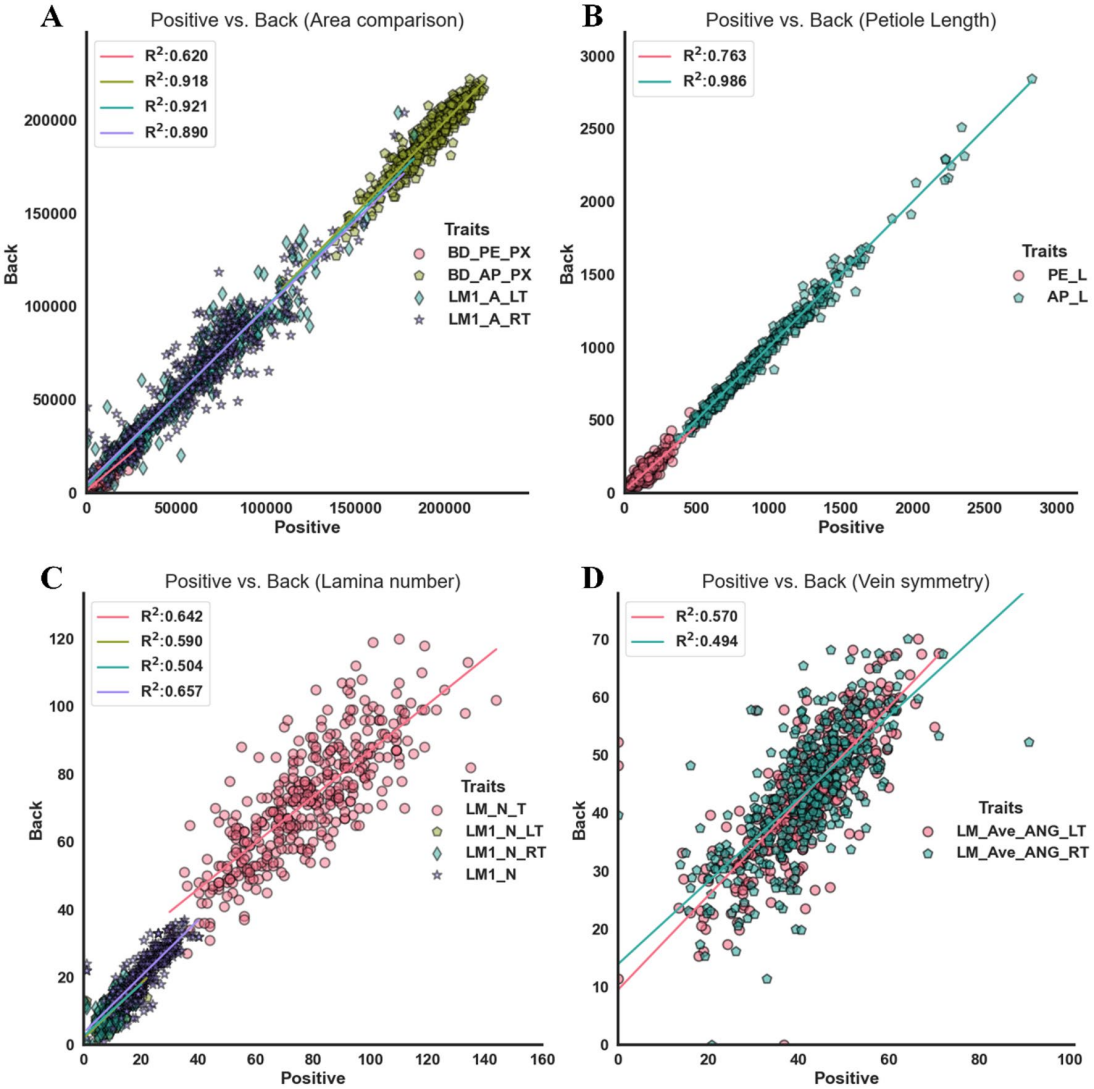
Vein architecture obtained using positive and back leaves. **A** Area comparison of semantic components between positive and back leaves. **B** Length comparison of the petiole. **C** Number comparison of lamina. **D** Average angle comparison of second-order veins

The computation traits of petiole and vein architecture extracted from positive and back images of the same leaf were very close. A grouping correlation analysis was also used to evaluate vein architecture of positive and back leaves (Fig. 10). The position sensitivity of the petiole related to the starting lamina was observed from Fig. 5A, indicating a low determination coefficient of BD_PE_PX between positive and back leaves (0.62) (Fig. 10A). LM1_A_LT and LM1_A_RT had high determination coefficients (0.921 and 0.890, respectively). The second-order veins were ordered in sequence along with the mid-rid, and obtained two angle lists (LM_ANG_List_LT and LM_ANG_List_RT). The average vein angles (LM_Ave_ANG_LT and LM_Ave_ANG_RT) of the left and right leaves were calculated to reveal the consistency of vein architecture between positive and back leaves, and the average vein angle was about 0.5.

### Evaluation of color traits of positive-back leaves

The positive and back leaves had significant phenotype differences that were relative to their physiological functions, especially color differences. Six color spaces (RGB, HSV, LAB, LUV, YCrCb, and CIELab) were used to represent the color traits of each semantic component of leaves. Each color space had three channels (18 channels). The mean and standard deviation of each channel was calculated. A feature set was constructed for each semantic component (each feature vector contained 18 mean color features) to identify the most important color feature of each semantic component. The orientation type (positive or back leaves) was used as a classification variable.

**Fig. 11:**
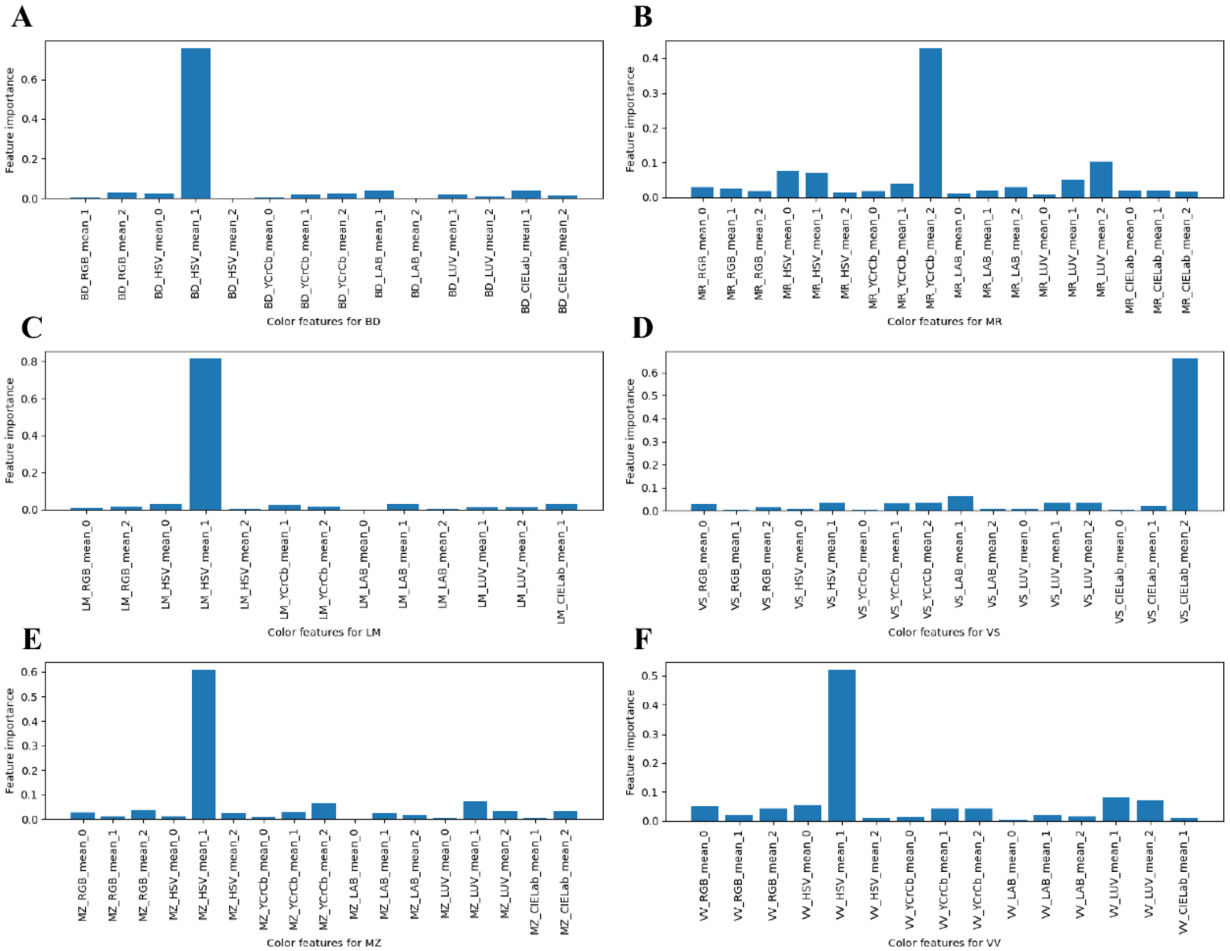
The feature importance of each semantic component. **A**–**F** BD, MR, LM, VS, MZ, and VV, respectively

A classification decision tree was used to determine the most important color features based on the classification variable (Fig. 11). All color features with importance scores greater than 0 were extracted from 18 color features. The important features of BD, MR, LM, VS, VV, and MZ were BD_HSV_mean_1 (75.672%), MR_YCrCb_mean_2 (42.939%), LM_HSV_mean_1 (81.538%), VS_CIELab_mean_2 (66.057%), VV_HSV_mean_1 (52.178%), and MZ_HSV_mean_1 (61.050%), respectively. The total importance scores of the first five features were 89.431%, 72.940%, 92.646%, 83.097%, 78.050%, and 81.460%, respectively, for each semantic component. LM had the most color difference between positive and back leaves. Laminas were the main photosynthetic regions, and their color features and difference were more accurate in evaluating physiological functions (chlorophyll content) of leaves. The color features of MR were complex, with a single feature explaining 42.939% at most. The petiole and vein network had a significant influence on the color representation of the whole leaf. Moreover, Hue (HSV) was the most stable and distinguishable color feature for BD, LM, VV, and MZ of positive and back leaves.

**Fig. 12:**
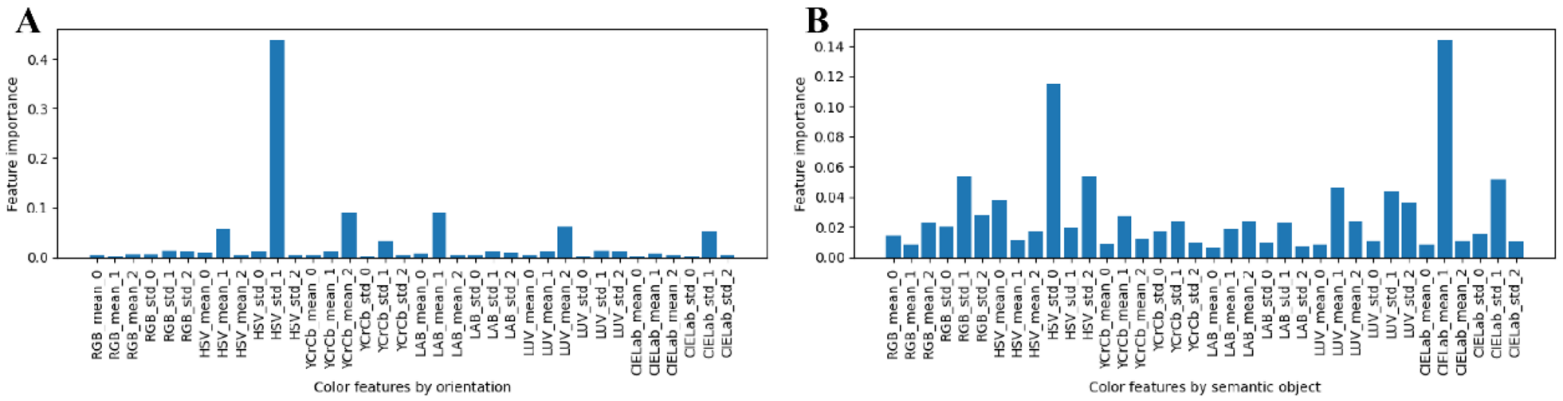
The feature importance of the whole leaves. **A** Orientation (postive-back leaf) as classification variable. **B** Semantic components as a classification variable

Color features of six semantic components were integrated into a feature set containing 216 color features to quantify the whole leaf color. The orientation (positive-back leaf) and semantic components were respectively used as classification variables to investigate the important scores of color features (Fig. 12A and B). The variance color features were found to play a crucial role, especially in using the orientation type as the classification variable. HSV_std_1 obtained the highest importance score (43.825%), followed by LAB_mean_1 (9.014%). The importance score of the first five-color features was 73.495% (Fig. 12A). The most important feature (CIELab_mean_1) when the semantic component was used as the classification variable explained just 14.398%. The importance score of the first five-color features was only 27.406%. These results suggested that the color differences among semantic components of lettuce leaves were significantly higher than that of positive-back leaves. Therefore, it was necessary to refine the color traits of the lettuce leaf by distinguishing semantic components.

### Clustering and classification of lettuce leaves

**Fig. 13:**
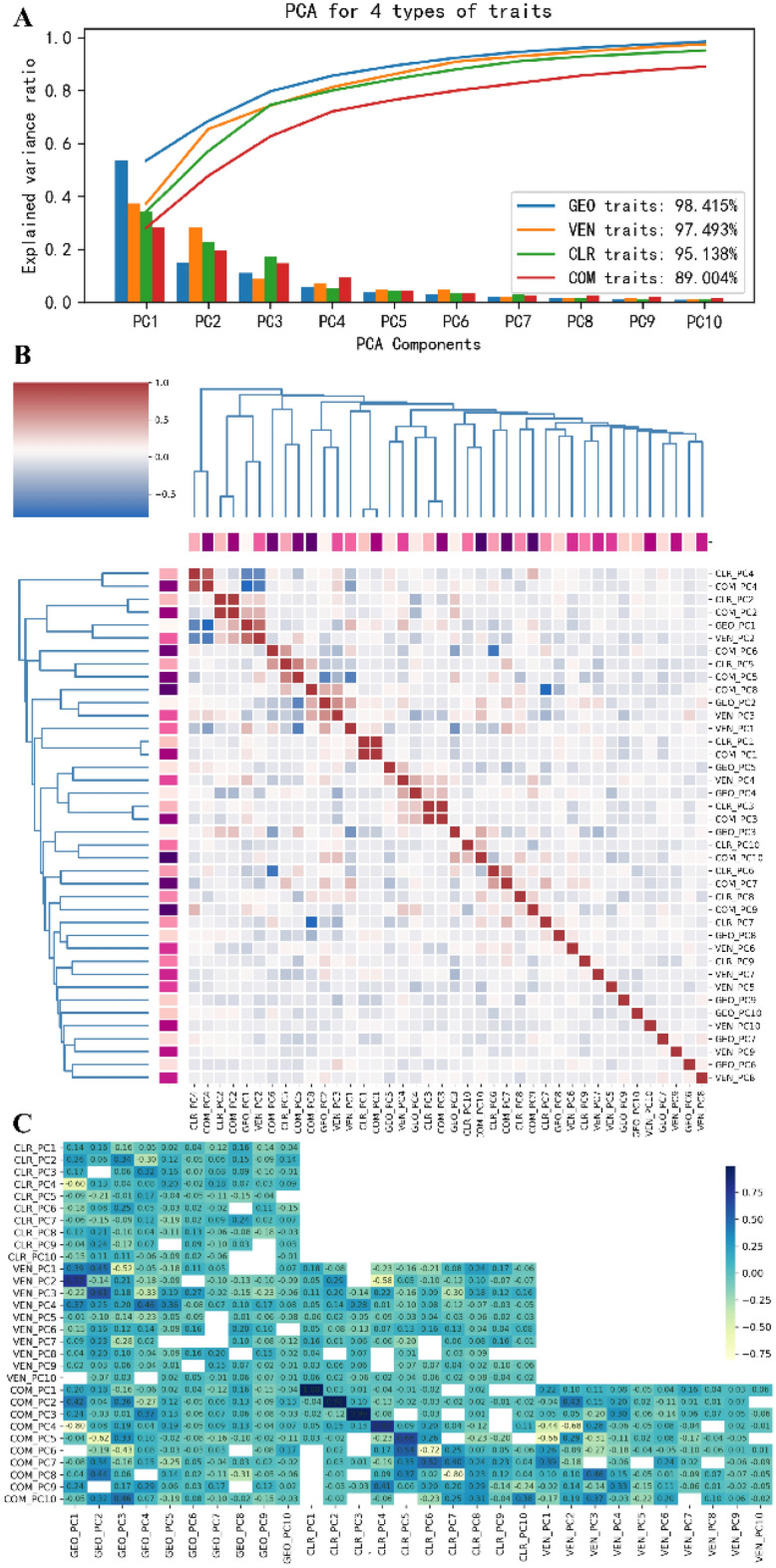
The principal components and correlation analysis of four types of traits. **A** Screen plot of the PCA model and the curves of cumulative explained variance. **B** Hierarchically-clustered heatmap for the correlation matrix of 40 PCs. **C** The correlation analysis for four types of PCs. Correlation coefficients less than 0.01 were not displayed

These semantic components of lettuce leaves contributed lots of accurate and quantitative traits that were difficult to be measured manually. Some traits had clear physiological and ecological significance, or are related to lettuce quality and yield, such as color differences of positive-back leaves, and area ratio of leaf veins, etc. Thus, these image-based traits were not only valuable for gene function analysis and mapping of lettuce varieties, but also could be used for classification and identification of lettuce leaves. The selection of important features were valuable for refining the description of lettuce leaf and its components. Principal component analysis (PCA) could be used for dimensionality reduction via fusion to reduce the number of features greatly. Both the single important feature or principal components could be used for leaf classification and grading. Herein, the feature sets were classified into four types [geometry-based (GEO, 30), venation-based (VEN, 20), color-based (CLR, 216), and combined (COM, 266)], then used for PCA analysis (400 lettuce varieties). The top 10 principal components of each feature set could explain 98.415%, 97.493%, 95.138%, and 89.004% of the overall variance, respectively (Fig. 13A). A hierarchically clustered heatmap was plotted to determine the correlation matrix among 40 PCs (Fig. 13B).

Each cluster merged the non-singleton cluster and its children by drawing a U-shaped link. The length of the two legs of the U-link indicated the distance between its child clusters. The correlation among the 10 PCs of each type of feature set was linearly independent. Moreover, several PC pairs were highly correlated among different feature sets. For instance, GEO_PC1 and VEN_PC2 had a significantly high correlation coefficient (CC) of 0.77. CLR_PC4 was negatively correlated with GEO_PC1 and VEN_PC2 (CCs were −0.60 and −0.58, respectively). The first three PCs of COM and CLR were highly positively correlated. The detailed correlation coefficients among different PCs are shown in Fig. 13C.

**Fig. 14:**
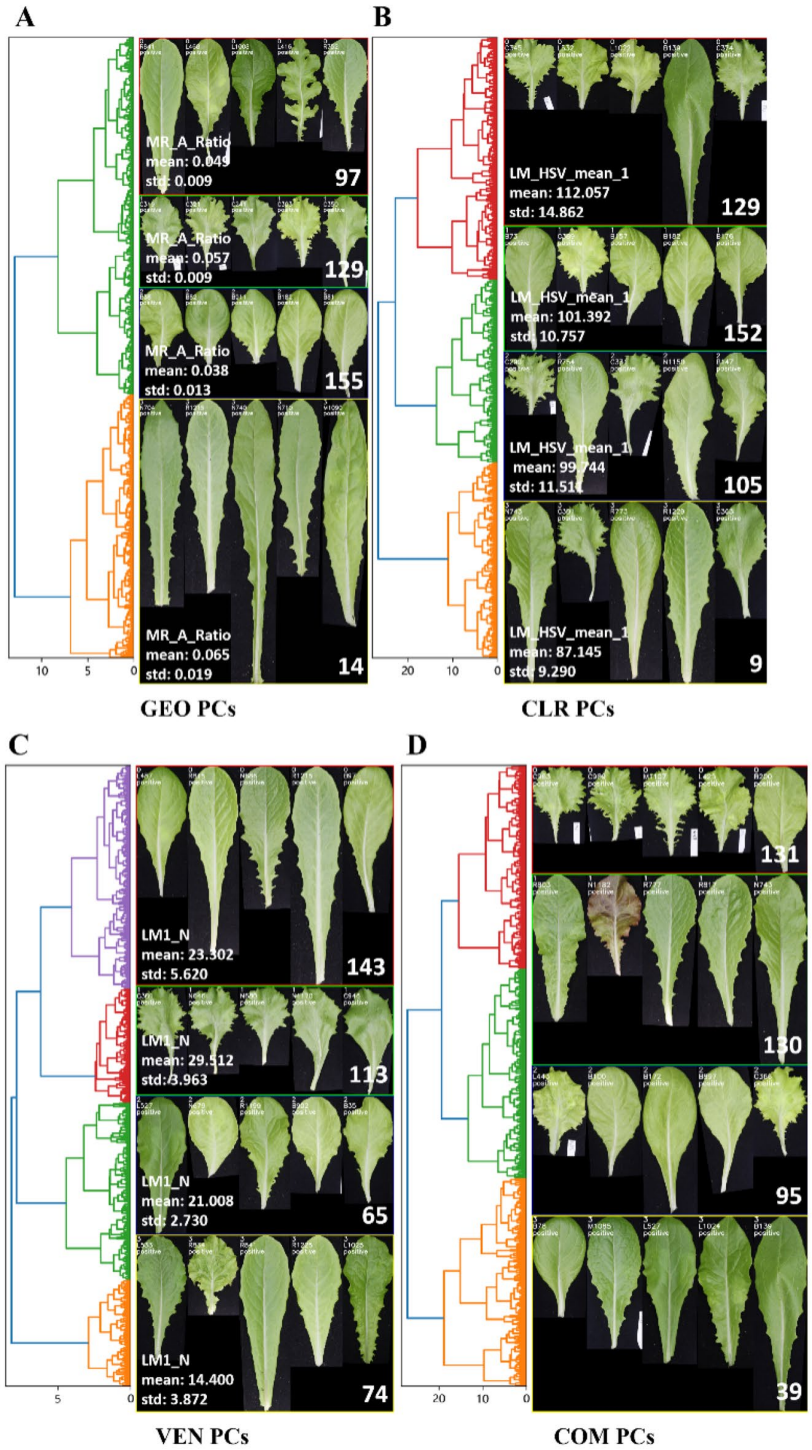
The hierarchical clustering and classification of positive leaves using the PCs of GEO (**A**), CLR (**B**), VEN (**C**), and COM (**D**). The leaves were clustered into four groups (the cluster id from 0 to 3, from top to bottom). Five leaves were randomly selected from each group and arranged in one row. The leaf number of each group is illustrated in the lower right corner, and statistical traits of each group are shown in the lower-left corner

Lettuce leaves could be clustered into different categories based on their feature set by minimizing the intra-class gap and maximizing the inter-class gap. Each type of lettuce leaves was assigned an individual label for further analysis. The hierarchical clustering based on Euclidean distance was conducted using four types of PCs (Fig. 14). For positive images (Fig. 14), four clusters were classified using the PCs of GEO, CLR, VEN, and COM traits. Five images were randomly selected from each classified leaf group for visualization. The leaves had significantly different cluster groups based on different feature sets. Three intuitive and meaningful traits (MR_A_Ratio, LM_HSV_mean_1, and LM1_N) from GEO, CLR, and VEN traits, respectively, were used to evaluate the clustered leaves. The mean and std of these traits were calculated based on each type of clustered leaves. The statistical results were intuitively displayed in the clustered leaf images. The GEO traits of the clustered leaves distinguished MR and petiole (Fig. 14A). The MR of cluster 3 occupied a large area of the leaf. The mean value of MR_A_Ratio represented the area ratio between the MR and the whole leaf. The mean value of cluster 3 (0.065) was higher than cluster 2 (0.038). The number of the first-order lamina (LM1_N) of cluster 1 (29.512) (Fig. 14B) was significantly higher than that of cluster 3 (14.4) (Fig. 14C). Similarly, the regions of first-order laminas of cluster 3 were large, and the second-order veins were well developed.

## Conclusion

Here we developed a phenotyping pipeline to extract and quantify multiple semantic components of leaves for different lettuce varieties. By fine definition and image annotation, semantic segmentation models were employed to automatically decompose lettuce leaves into six types of semantic components. Through the normalization operations, leaves of great differences in size and orientation were rotated and scaled to the same level for trait quantification and comparison. The positive-back lettuce leaves were also for the first time used to evaluate the accuracy of semantic segmentation and their essential difference from the geometry, color, and venation perspectives. These semantic components contribute a large number of traits that are difficult to be measured manually and are helpful to discover the statistical differences of lettuce leaves for leaf classification and identification. The proposed method can be improved in segmentation accuracy by supplementing richer annotated leaves and be extended for leaf phenotyping of other crops.

## Supplementary Information

**Additional file 1:** **Table S1.** Model evaluation for six semantic components of lettuce leaves. **Table S2.** Geometry traits of lettuce leave. **Table S3.** Vein architecture traits of lettuce leaves.

## Data Availability

The all data sets used analyzed in this study are available from the corresponding author on reasonable request.
