# Supplementary material for: Quantitative phenotyping and evaluation for lettuce leaves of multiple semantic components
Source: Plant Methods. 2022 Apr 25;18:54. doi: 10.1186/s13007-022-00890-2 (PMC9036747; doi:10.1186/s13007-022-00890-2)
Supplement: Supplementary file 1 — Additional file 1: Table S1. Model evaluation for six semantic components of lettuce leaves. Table S2. Geometry traits of lettuce leave. Table S3. Vein architecture traits of lettuce leaves. [file 13007_2022_890_MOESM1_ESM.docx]

## Supplementary material

**Supplementary Table 1. Model evaluation for six semantic components of lettuce leaves**

|  | **Blade** | **Mid-rid** | **Veins** | **Venation** | **Lamina** | **Marginal zone** |
| --- | --- | --- | --- | --- | --- | --- |
| Training IoU | 0.99528 | 0.91782 | 0.78774 | 0.80342 | 0.92387 | 0.85704 |
| Training Loss | 0.00440 | 0.04902 | 0.14926 | 0.14380 | 0.06425 | 0.11502 |
| Validation IoU | 0.99551 | 0.93608 | 0.82023 | 0.83660 | 0.91715 | 0.85234 |
| Validation Loss | 0.00405 | 0.03868 | 0.12783 | 0.11956 | 0.07314 | 0.12855 |
| Testing mIoU | 0.99617 | 0.90176 | 0.70089 | 0.74428 | 0.83299 | 0.71872 |
| Testing mF1 | 0.99808 | 0.94818 | 0.82378 | 0.85299 | 0.90857 | 0.83623 |

**Supplementary Table 2. Geometry traits of lettuce leaves**

| **Index** | **Abbreviation** | **Description** | **Unit** |
| --- | --- | --- | --- |
| 1 | BD_A | Total leaf area | ${pixel}^{2}$ |
| 2 | BD_W | Leaf width | $pixel$ |
| 3 | BD_L | Leaf length | $pixel$ |
| 4 | BD_HWL | Ratio between leaf width and length |  |
| 5 | MR_A | Mid-rid area | ${pixel}^{2}$ |
| 6 | MR_L | Mid-rid length | $pixel$ |
| 7 | MR_W | Mid-rid width | $pixel$ |
| 8 | VS_A | Area of the second-order veins | ${pixel}^{2}$ |
| 9 | VS_CHA | Convex area of the second-order veins | ${pixel}^{2}$ |
| 10 | VS_N | Number of the second-order veins |  |
| 11 | LM_A | Area of the first-order laminas | ${pixel}^{2}$ |
| 12 | LM_CHA | Convex area of the first-order laminas | ${pixel}^{2}$ |
| 13 | LM_MA | Max area of the first-order laminas | ${pixel}^{2}$ |
| 14 | LM_N | Number of the first-order laminas |  |
| 15 | VV_A | Area of the vein venation | ${pixel}^{2}$ |
| 16 | VV_CHA | Convex area of the vein venation | ${pixel}^{2}$ |
| 17 | MZ_A | Area of the marginal zone | ${pixel}^{2}$ |
| 18 | MZ_HN | Hole number of the marginal zone |  |
| 19 | MZ_HA | Hole area of the marginal zone | ${pixel}^{2}$ |
| 20 | MR_A_Ratio | Area ratio between the mid-rid and the leaf |  |
| 21 | VS_A_Ratio | Area ratio between the second-order veins and the leaf |  |
| 22 | VV_A_Ratio | Area ratio between the vein venation and the leaf |  |
| 23 | LM_A_Ratio | Area ratio between the laminas and the leaf |  |
| 24 | MZ_A_Ratio | Area ratio between the marginal zone and the leaf |  |
| 25 | MR_VS_A_Ratio | Area ratio: (MR_A+VS_A)/BD_A |  |
| 26 | MR_VV_A_Ratio | Area ratio: (MR_A+VV_A)/BD_A |  |
| 27 | VS_VV_A_Ratio | Area ratio: (VS_A+VV_A)/BD_A |  |
| 28 | EVA_MR_VS_2_VV | Area ratio: (MR_A+VS_A)/VV_A |  |
| 29 | EVA_LM_VV_2_BD | Area ratio: (LM_A+VV_A)/BD_A |  |
| 30 | EVA_MZ_LM_2_BD | Area ratio: (MZ_A+LM_A)/BD_A |  |

**Supplementary Table 3. Vein architecture traits of lettuce leaves**

| **Index** | **Abbreviation** | **Description** | **Unit** |
| --- | --- | --- | --- |
| 1 | LM_N_T | Total number of laminas |  |
| 2 | BD_PE_PX | Area of petiole | ${pixel}^{2}$ |
| 3 | BD_AP_PX | Area from petiole to the apex | ${pixel}^{2}$ |
| 4 | MR_PE_PX | Area of petiole in the mid-rid region | ${pixel}^{2}$ |
| 5 | MR_AP_PX | Area from petiole to apex in the mid-rid region | ${pixel}^{2}$ |
| 6 | PE_L | Length of petiole | $pixel$ |
| 7 | AP_L | Length from petiole to the apex | $pixel$ |
| 8 | BD_PE_AP_AR | Area ratio between petiole and apex |  |
| 9 | MR_PE_AP_AR | Area ratio between petiole and apex in the mid-rid region |  |
| 10 | PE_ AP_LR | Length ratio between apex and petiole |  |
| 11 | LM1_N_LT | Number of the first-order lamina of the left side |  |
| 12 | LM1_N_RT | Number of the first-order lamina of the right side |  |
| 13 | LM1_N | Number of the first-order lamina |  |
| 14 | LM1_NR | $R1=LM1\_N\_LT/ LM1\_N\_RT$  $R2=LM1\_N\_RT/ LM1\_N\_LT$  $R = min(R1, R2)$ |  |
| 15 | LM1_A_LT | Area of the first-order laminas on the left side |  |
| 16 | LM1_A_RT | Area of the first-order laminas on the right side |  |
| 17 | LM1_AR | $R1= LM1\_A\_LT / LM1\_A\_RT$  $R2= LM1\_A\_RT / LM1\_A\_LT$  $R = min(R1, R2)$ |  |
| 18 | LM_ANG_List_LT | Angle list of the left first-order lamina | $degree(^{\circ})$ |
| 19 | LM_ANG_List_RT | Angle list of the right first-order lamina | $degree(^{\circ})$ |
| 20 | LM_Ave_ANG_LT | Average angle of the left first-order lamina | $degree(^{\circ})$ |
| 21 | LM_Ave_ANG_RT | Average angle of the right first-order lamina | $degree(^{\circ})$ |
| 22 | LM_NR_ANG | Angle ratio between the left and right first-order lamina, and [less](javascript:;) [than](javascript:;) [or](javascript:;) [equal](javascript:;) [to](javascript:;) 1.  $R1= LM\_Ave\_ANG\_LT / LM\_Ave\_ANG\_RT$  $R2= LM\_Ave\_ANG\_RT / LM\_Ave\_ANG\_LT$  $R = min(R1, R2)$ |  |
